# Astrocytes evoke a robust IRF7-independent type I interferon response upon neurotropic viral infection

**DOI:** 10.1186/s12974-023-02892-w

**Published:** 2023-09-22

**Authors:** Loreen Weichert, Henning Peter Düsedau, David Fritzsch, Sarah Schreier, Annika Scharf, Martina Grashoff, Kristin Cebulski, Kristin Michaelsen-Preusse, Christian Erck, Stefan Lienenklaus, Ildiko Rita Dunay, Andrea Kröger

**Affiliations:** 1 Molecular Microbiology Group, Institute of Medical Microbiology and Hospital Hygiene, Otto-von-Guericke-University Magdeburg, 39120 Magdeburg, Germany; 2 Innate Immunity and Infection, Helmholtz Centre for Infection Research, 38124 Braunschweig, Germany; 3 Institute of Inflammation and Neurodegeneration, Otto-von-Guericke-University Magdeburg, 39120 Magdeburg, Germany; 4 Division of Cellular Neurobiology, TU Braunschweig, 38106 Braunschweig, Germany; 5 Cellular Proteome Research, Helmholtz Centre for Infection Research, 38124 Braunschweig, Germany; 6 Institute for Laboratory Animal Science, Hanover Medical School, 30625 Hannover, Germany; 7 Health Campus Immunology, Infectiology, and inflammation (GC-I3), Magdeburg, Germany; 8 Center for Behavioral Braun Science (CBBS), 39106 Magdeburg, Germany

**Keywords:** Innate immunity, TBEV, LGTV, Astrocytes, Type I interferon, IRF7

## Abstract

**Background:**

Type I interferons (IFN-I) are fundamental in controlling viral infections but fatal interferonopathy is restricted in the immune-privileged central nervous system (CNS). In contrast to the well-established role of Interferon Regulatory Factor 7 (IRF7) in the regulation of IFN-I response in the periphery, little is known about the specific function in the CNS.

**Methods:**

To investigate the role for IRF7 in antiviral response during neurotropic virus infection, mice deficient for IRF3 and IRF7 were infected systemically with Langat virus (LGTV). Viral burden and IFN-I response was analyzed in the periphery and the CNS by focus formation assay, RT-PCR, immunohistochemistry and in vivo imaging. Microglia and infiltration of CNS-infiltration of immune cells were characterized by flow cytometry.

**Results:**

Here, we demonstrate that during infection with the neurotropic Langat virus (LGTV), an attenuated member of the tick-borne encephalitis virus (TBEV) subgroup, neurons do not rely on IRF7 for cell-intrinsic antiviral resistance and IFN-I induction. An increased viral replication in IRF7-deficient mice suggests an indirect antiviral mechanism. Astrocytes rely on IRF7 to establish a cell-autonomous antiviral response. Notably, the loss of IRF7 particularly in astrocytes resulted in a high IFN-I production. Sustained production of IFN-I in astrocytes is independent of an IRF7-mediated positive feedback loop.

**Conclusion:**

IFN-I induction in the CNS is profoundly regulated in a cell type-specific fashion.

## Introduction

Viral infections of the CNS are rather rare but devastating. Most of the neurotropic flaviviruses lead to subclinical diseases in humans [1]. Although the strict regulation of immune cell entry into the immune-privileged CNS protects the brain, conversely, leaves it particularly vulnerable to infections. Due to the critical functions as well as longevity of resident brain cells, it is necessary to prevent potential neuronal damage by inflammation and cytotoxic cells [2]. Still, most of the neurotropic viral infections are defeated by virus-specific innate and acquired host immune responses in the CNS.

The IFN-I system is the first line of defense of the body against viral infections. IFN-I response is triggered by the recognition of pathogen-associated molecular patterns (PAMPs) by several host pattern recognition receptors (PRRs) and is biphasic. Recognition of viral RNA by PRRs occurs by cytoplasmic retinoic acid-inducible gene (RIG-I)-like receptors (RLRs) or by toll-like receptors (TLRs) [3–7]. PRR signaling results in the activation of the transcription factors NF-κB or IRF3 which induce IFN-Is. Upon binding of its heterodimeric receptor IFNAR, a transcriptional activation of IFN-stimulated genes (ISGs) is induced which with its products collaboratively interfering with viral replication and spread on several levels. IRF7 is an ISG that upon activation induces a positive feedback loop for high IFN-I induction [8]. The main producers of systemic IFN-I upon viral infection are the plasmacytoid dendritic cells (pDCs). Induction of IFN-I is mainly mediated by a TLR-dependent pathway, and crucially dependent on IRF7 which is constitutively expressed in pDCs [9, 10]. Thus, systemic or pDC-dependent production of IFN-I relies on IRF7 [9, 11].

IFN-I response upon viral infection in the periphery leads to inhibition of viral replication and spread, dampens viremia and thereby reduces neuroinvasion. In the CNS intrinsic and indirect IFN-I-based mechanisms restrict local viral replication. In the absence of the IFN-I receptor mice are highly vulnerable to encephalitis caused by vector-borne flaviviruses like West Nile virus (WNV) [12], Yellow fever virus [13], TBEV [14] and Japanese encephalitis virus [15].

IFN-I response in neuroectodermal cells is essential to protect mice from vesicular stomatitis virus or Langat virus (LGTV) infection [14, 16], indicating that local IFN response in the brain is essential. Although all cells of the CNS are able to react to IFN-I, it is still unclear which cells represent the main source of IFN production. While peripheral tissues rely on pDCs as the major IFN-I-producing cells, the uninfected brain is largely devoid of this cell type, indicating that CNS-resident cells must be responsible for the production of substantial amounts of IFN-I upon virus infection. Studies using influenza virus, La Crosse virus and vesicular stomatitis virus (VSV) showed that parenchymal cells of the CNS such as microglia, neurons and astrocytes are able to produce IFN-I [17, 18].

Our previous studies indicated a specific importance of locally produced IFN-I in the brain upon infection with neurotropic LGTV, an attenuated member of the TBEV family [14, 19]. IRF7 was once termed as the master regulator of IFN-I production. However, in contrast to the well-established role of IRF7 in the periphery its specific role in the CNS remains elusive. Therefore, we aimed to uncover the mechanism of IFN-I induction, and the differences between peripheral versus local induction in the brain, focusing on brain-resident cells. Taking advantage of IRF7- and IRF3-deficient mice, our data underline that IRF3 and IRF7 are dispensable for the survival of LGTV-infected mice although IRF7 is needed for IFN-I production in the periphery. In contrast, in the CNS IRF7 deficiency leads to high expression levels of IFN-I upon infection, indicating differential regulation of IFN-I expression in the brain. Intrinsic antiviral response by IRF7 is mediated specifically in astrocytes but not in neurons, pointing towards a cell type-specific regulation of IFN-I induction in a cell type-specific manner in the CNS.

## Methods

### Ethics statement

All animal experiments were performed in compliance with the German animal welfare law (TierSchG BGBl. S. 1105; 25.05.1998). The mice were housed and handled in accordance with good animal practice as defined by FELASA. All animal experiments were approved by the Lower Saxony State Office of Consumer Protection and Food Safety under permit number AZ 33.9-42,502-04-11/0528 or by the Landesverwaltungsamt Sachsen-Anhalt AZ 42502-2-1344, University of Magdeburg.

### Mice and viral infection

Specific pathogen-free (SPF) C57BL/6 (WT) mice were obtained from ENVIGO. *Irf3*^*−/−*^ (Irf3^tm1Ttg^), *Irf7*^*−/−*^ (Irf7^tm1Ttg^) and *Ifnβ*^+*/Δluc*^ (Ifnb1^tm2.2Lien^) mice on C57BL/6 background were bred under SPF conditions at the Helmholtz Centre for Infection Research in Braunschweig or the Otto von Guericke University Magdeburg, Germany. LGTV strain TP21 (G. Dobler) was propagated in Vero B4 cells. Titers were determined by focus forming assays on Vero B4 cells [20]. Six- to ten-week-old mice were infected intraperitoneally (i.p.) with 10^4^ focus forming units (FFU) in 100 µl 1 × PBS. For intracranial infections (i.c.), mice were anesthetized with ketamine (100 mg/kg body weight) supplemented with xylazine (5 mg/kg body weight) from CD-Pharma. Mice were injected with indicated FFU of LGTV in 20 μl 1 × PBS. Animals were killed at humane endpoints and perfused with 20 mL of PBS.

### In vivo imaging

For in vivo imaging, *Ifnβ*^+*/Δluc*^, *Irf3*^*−/−*^* Ifnβ*^+*/Δluc*^ and *Irf7*^*−/−*^* Ifnβ*^+*/Δluc*^ albino (Tyr^c−2 J^) C57BL/6 mice were inoculated intravenously with D luciferin (150 mg/kg in PBS, SynChem OHG) and anesthetized using isoflurane (Baxter). Animals were monitored in the IVIS 200 imaging system (CaliperLS) and relative intensity of emitted light is represented as pseudocolor image ranging from red (high) to blue (low). Signals are displayed as photon flux, quantified as radiance p/sec/cm3/sr (Living Image 4.2 software; Caliper).

### RNA extraction and quantitative RT-PCR

For quantitative RT-PCR of RNA expression levels, mouse organs were homogenized in peqGOLD TriFast (PeqLab, #30-2010) using the homogenizer Fast Prep 24 (MP). RNA was isolated according to the manufacturer’s instruction. QIAamp Viral RNA Mini Kit (Cat.No. 52904 Fa. QIAGEN) was used for RNA extraction from serum. cDNA synthesis from RNA was done with RNA First strand cDNA synthesis kit (GE, Healthcare) and quantitative qRT-PCR was carried out by using KAPA SYBR FAST Universal Master Mix (PeqLab, # 07-KK4600-02). Following murine primers were used for qPCR: CCL2 (forward primer 3′-GCC CCA CTC ACC TGC TGC TA-5′, reverse primer 3′-TTT ACG GGT CAT CAC ATT CAA-5′), TNF α (forward primer 3′- GAA CTG GCA GAA GAG GCA CT-5′, reverse primer 3′- AGG GTC TGG GCC ATA GAA CT-5′), IFN-β (forward primer 3′- CAC AGG CCA TGA AGG AAG AT′-5, reverse primer 3′- CAT TAC CTG AAG GCC AAG GA-5), pan-IFN-α (forward primer 3′-CTA GAC TCA TTC TGC AAT G-5′, reverse primer 3′-TCC TCA CAG CCA GCA GG-5′). LGTV RNA was determined by KAPA probe FAST qPCR kit using LGTV NS3 based Taq-Man assay (forward primer 5′AAC GGA GCC ATA GCC AGT GA-3′, reverse primer 5′AAC CCG TCC CGC CAC TC-3′, probe FAM-AGA GAC AGA TCC CTG ATG GMG B) with a sensitivity of 10 viral copies. All samples were measured by Light Cycler 480 II (Roche), normalized to gene expression of β-actin and calculated by the ΔΔCT method.

### In vitro differentiation of bone marrow-derived cells

For in vitro generation of BMDDCs or BMDM, bone marrow-derived cells were isolated by flushing the femur and tibia of mice with RPMI 1640 medium (Gibco) supplemented with 10% FCS, 10 mM HEPES, 1 mM sodium pyruvate, 2 mM GlutaMAX (Life Technologies), 100 U/mL penicillin (Life Technologies), 100 μg/mL streptomycin (Life Technologies) and 0.1 mM 2 ME. Bone marrow cells were cultivated for 8 days at a density of 1.5 × 10^6^/mL supplemented with 100 ng/mL GM-CSF (adherent macrophages and mDC suspension cultures) or Flt-3L (pDC suspension cultures). The medium was changed once by replacing two-thirds of the medium with the fresh cytokine-supplemented medium. Macrophages as well as cell suspensions of mDCs and pDCs were infected for 1 h, 37 °C with LGTV, MOI 0.5. Supernatants of all cell cultures were collected 24/48/72 h post-infection and tested for viral replication and IFN-I production.

### Primary cell isolation

Primary embryonic hippocampal neurons from C57Bl/6 WT and *Irf7*^*−/−*^ mice were prepared at embryonic day 18 as described previously [21]. 70,000 cells were seeded on poly-L-lysin-coated cover slips and differentiated for 2 weeks in Neurobasal medium (Invitrogen) supplemented with N2 supplement (Invitrogen), 2% B27 (Invitrogen) and 0.5 mM GlutaMax at 37 °C, 5% CO_2_ and 95% humidity. Differentiated neuronal cells were infected with LGTV (MOI 0.001) and viral replication was analyzed by focus forming assay (FFA).

For astrocyte cultures postnatal mice (P1–P3) were killed by decapitation and whole brains were homogenized through a 100 µm strainer into ice-cold HBSS containing calcium and magnesium (Gibco, #24020). Cell pellets were dissolved into DMEM (Gibco, # 11995) supplemented with 10% FCS (Merck Millipor, # S0615), 1% N-2 supplement (Gibco, # 17502), 0.5 mM GlutaMax and 0.1 U/mL penicillin and 0.1 ug/mL streptomycin and seeded at a density of 2.5 × 10^4^ cells/ cm^2^ into poly D-Lysin-coated culture flasks. Medium was changed 1 day post isolation and later renewed every 7 or 8 days. Microglia and oligodendrocyte precursors were removed by shaking, 600 rpm, 1 h, RT. For infection assays, 70,000 cells were seeded on poly D-Lysin coated cover slips and infected with LGTV (MOI 0.1) or transfected with different concentrations of poly (I:C) (Invivogen). PeqFECT (VWR PeqLab) was used for transfection according to the manual instructions. Kinetics of viral replication and IFN-I secretion were analyzed by FFA and IFN-I bioassay.

### Focus forming assay and virus titration

Virus titers in supernatants were quantified by focus forming assay as described previously [20]. Briefly, serial dilutions of LGTV samples were added on Vero B4 cells for 1 h. Inoculum was removed and cells were overlayed with 1.5% Avicel RC/CL in 1 × DMEM supplemented with FCS, penicillin/streptomycin and glutamine. After 72 h, cells were fixed with 6% paraformaldehyde and permeabilized by 1 × PBS, 0.5% Triton X 100, 20 mM glycine. LGTV foci were stained with mouse anti-TBEV E MoAb 19/1786 [22] and secondary rabbit anti-mouse horseradish peroxidase (HRP)-conjugated antibody (Jackson, no. 315-035) in 1 × PBS supplemented with 10% FCS. LGTV-positive foci were visualized by TrueBlue staining (KPL, Gaithersburg).

### Flow cytometry

Brains were harvested from mice upon perfusion with 60 mL 1 × PBS and subsequently used for cell isolation as described previously [23]. In brief, brains were homogenized in a buffer containing HBSS (Gibco), 13 mM HEPES (pH 7.3, Thermo Fisher Scientific) and 0.68% glucose before sieving through a 70 µm cell strainer. The homogenate was fractioned on a discontinuous 30–70% Percoll gradient (GE Healthcare). Immune cells were collected from the 30/70% Percoll interphase, washed in PBS and immediately processed for subsequent flow cytometry. For flow cytometric analysis of brain immune cells, freshly isolated cells were first incubated with Zombie NIR™ fixable dye (BioLegend) for live/dead discrimination in combination with anti-FcγIII/II receptor antibody (clone 93) to prevent unspecific binding of antibodies. Thereafter, cells were further stained with the following fluorochrome-conjugated antibodies against cell surface markers in FACS buffer (PBS containing 2% fetal bovine serum and 0.1% sodium azide): eFluor™ 450-CD45 (clone 30-F11), FITC-CD4 (clone GK1.5), PerCP/Cy5.5-Ly6C (clone HK1.4), PE/Cy5-Ly6G (clone 1A8), PE/Cy7-CD11c (clone N418), and APC-CD11b (clone M1/70) (all purchased from eBioscience™); Brilliant Violet™ 510-B220 (CD45R) (clone RA3-6B2), Brilliant Violet™ 605-NK1.1 (clone PK136), Brilliant Violet™ 711-MHC Class II (I-A/I-E) (clone M5/114.15.2), PE-CD3 (clone 17A2), PE/Dazzle594™-CD206 (clone C068C2), AlexaFluor^®^ 700-CD8a (clone 53–6.7) (all purchased from BioLegend). Cells were acquired using an Attune NxT Flow Cytometer (ThermoFisher) equipped with 405, 488, 561, and 633 nm lasers. Obtained data were analyzed using FlowJo software (version 10.8.1, FlowJo LLC, USA). Fluorescence Minus One (FMO) controls were used to determine the level of autofluorescence.

### IHC

Immunohistological analyses were performed on mouse brains harvested after cardiac perfusion with 1 × PBS. Samples were fixated in 4% Rotifix (Roth), 24 h, transferred into 30% sucrose in 1 × PBS, 24 h, RT and then frozen in TissueTek O.C.T. compound (Sakura) and stored at − 80 °C. 30 µm sagittal brain slices were obtained by cryotome sectioning (Frigomobil, Leica) and staining procedure was done in free-floating whole-brain sections. Brain slices were blocked 1 h, RT in 1 × PBS, 1% BSA, 0.2% Triton and 10% goat serum and incubated in primary antibody solution overnight at 4 °C. Samples were stained with monoclonal TBEV E antibody (MoAb19/1786) [22], monoclonal rabbit anti-GFAP (Sigma), monoclonal guinea pig anti-NeuN (Synaptic Systems), polyclonal rabbit anti-IBA-1 (Synaptic System) and polyclonal rabbit anti-cleaved caspase 3 [Asp175] (Cell Signaling Technologies) antibody. Subsequently, slices were incubated with DAPI (1 µg/mL), secondary anti-mouse, anti-guinea pig, or anti-rabbit antibodies conjugated with FITC, Cy3, or Cy5 (Jackson ImmunoResearch) for 1 h at RT. Representative pictures were taken by LSM5 (Zeiss) microscope at 20 × and 40 × magnification and are shown for each regions.

### IFN assay

To determine the amount of IFN, IFN-sensitive epithelial cells from Mx2 Luc reporter mice were treated with supernatant or serum as described previously [24]. A standard curve for the calculation of IFN concentrations was obtained by treating cells with serial dilutions of IFN-β. Cells were lysed with Reporter Lysis buffer (Promega) and luciferase was measured by Berthold Luminometer Luma 9507.

### Statistical analysis

Relative body weight was compared by by Mann–Whitney test. Data from flow cytometry and RT-qPCR were compared by Student’s *t* test using GraphPad Prism 7 (GraphPad Software, CA, USA). In all cases, results are presented as arithmetic means and were considered significant, with a *P *value of < 0.05.

## Results

### IRF7 is dispensable for infection with neurotropic LGTV

**Fig. 1:**
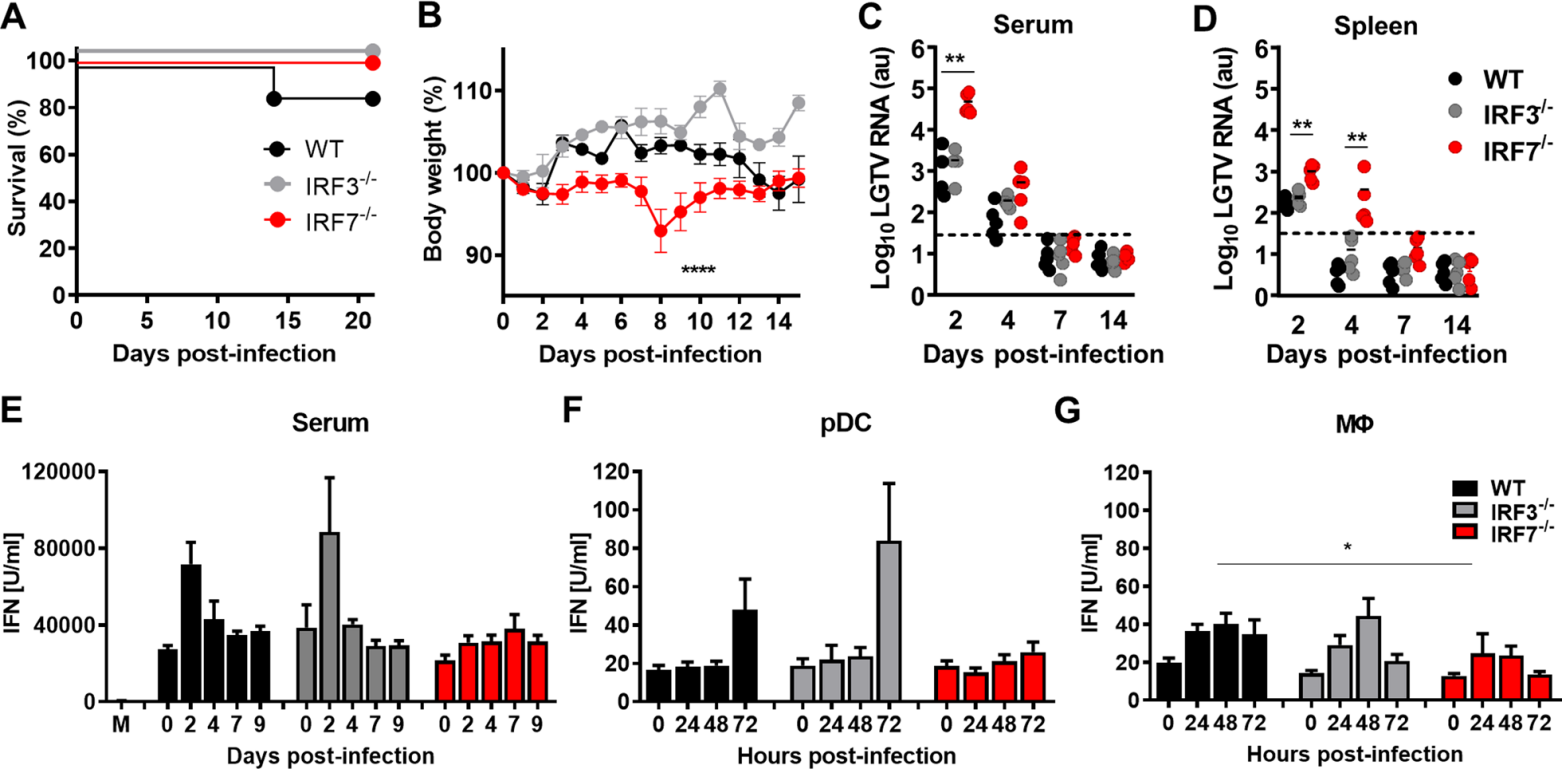
IRF7 is dispensable for the survival of LGTV infection. WT, *Mavs*^*−/−*^, *Irf3*^*−/−*^ and *Irf7*^*−/−*^ mice were intraperitoneally infected with 10^4^ FFU of LGTV (*n* = 10). **A** Survival and **B** body weight were monitored. Data represent mean with SEM of *n* ≥ 10 in each group per time point. Viral burden was determined by quantitative RT-PCR in the serum (**C**) and spleen (**D**). The dotted line represents the detection limit. IFN-I levels were determined by bioassay in serum (**E**) or in the supernatant of infected pDCs (F) or MØ (G). Asterisks indicate statistical significance calculated by Mann–Whitney test (**A**, **C**–**G**) or Student’s *t* test (**B**), *p<0.05, **p<0.01. Results are representative for at least two independent experiments

It was previously shown that *Irf7*^*−/−*^ mice are highly vulnerable to viral infections [11]. To assess the specific role of IRF3 and IRF7 in initiating an antiviral response in the periphery versus the CNS in neurotropic virus infection, we infected WT, *Irf3*^*−/−*^ and *Irf7*^*−/−*^ mice intraperitoneally with LGTV (Fig. 1). Surprisingly, similar to WT mice *Irf3*^*−/−*^ and *Irf7*^*−/−*^ animals survived the infection with no obvious signs of disease (Fig. 1A). However, *Irf7*^*−/−*^ mice showed a mild transient drop of body weight on day 8 post-infection (Fig. 1B). These data indicate that IRF3 and IRF7 are dispensable for survival following neurotropic LGTV infection.

Since arboviral infections are often associated with viremia, viral titers in serum and spleen were analyzed by quantitative real-time RT-PCR. A significantly elevated amount of viral RNA was found in serum of *Irf7*^*−/−*^ mice 2 days post-infection when compared to WT and *Irf3*^*−/−*^ mice (Fig. 1C). By day 4 post-infection viral RNA levels declined in *Irf7*^*−/−*^ mice and only slight differences could be detected between different groups, by day 7 post-infection no viral RNA levels are detectable. In spleens significant differences in viral load were detectable on 2 and 4 days post-infection in *Irf7*^*−/−*^ mice when compared to WT animals (Fig. 1D), further no viral RNA was detectable from day 7 post-infection. Taken together, IRF7 is dispensable for the survival of LGTV-infected mice but has a crucial function in early restriction of systemic viral replication.

LGTV replication is controlled by IFN-Is [14, 19]. Since IRF7 is essential for the induction of IFN-I via the virus-activated, MyD88-independent and the TLR-MyD88-dependent pathways [9], we determined the impact of IRF7 on the peripheral IFN-I response upon LGTV infection. Elevated systemic levels of IFN-I were detected in the serum of WT and *Irf3*^*−/−*^ mice 2 days post-infection, which declined by day 4 post-infection (Fig. 1E). In contrast, *Irf7*^*−/−*^ mice showed no increase of serum IFN-I levels upon infection. IRF7 has been identified as the master regulator of IFN-I induction in pDCs [9]. To determine which cell types are responsible for the reduced IFN-I response, we analyzed IFN-I level in bone marrow-derived pDCs, and macrophages (MØ) upon LGTV infection. The IFN-I production of pDCs was clearly dependent on IRF7, since no IFN-I was detectable in the supernatant of IRF7-deficient cells (Fig. 1F). The ability of macrophages to produce IFN-I was reduced in *Irf7*^*−/−*^ cells but not completely abolished (Fig. 1G). The inability to produce IFN-I was independent of the infection rate since *Irf7*^*−/−*^ pDCs, mDCs and macrophages showed similar infection rates upon challenge with LGTV (data not shown). Interestingly, IRF3-deficient macrophages were highly infected by LGTV in comparison to WT and *Irf7*^*−/−*^ cells, but no differences in IFN-I production was determined. Taken together our data indicate that IRF7 is important to mount peripheral IFN-I responses during LGTV infection in vivo.

### IRF7 limits LGTV replication and dissemination in the CNS

**Fig. 2:**
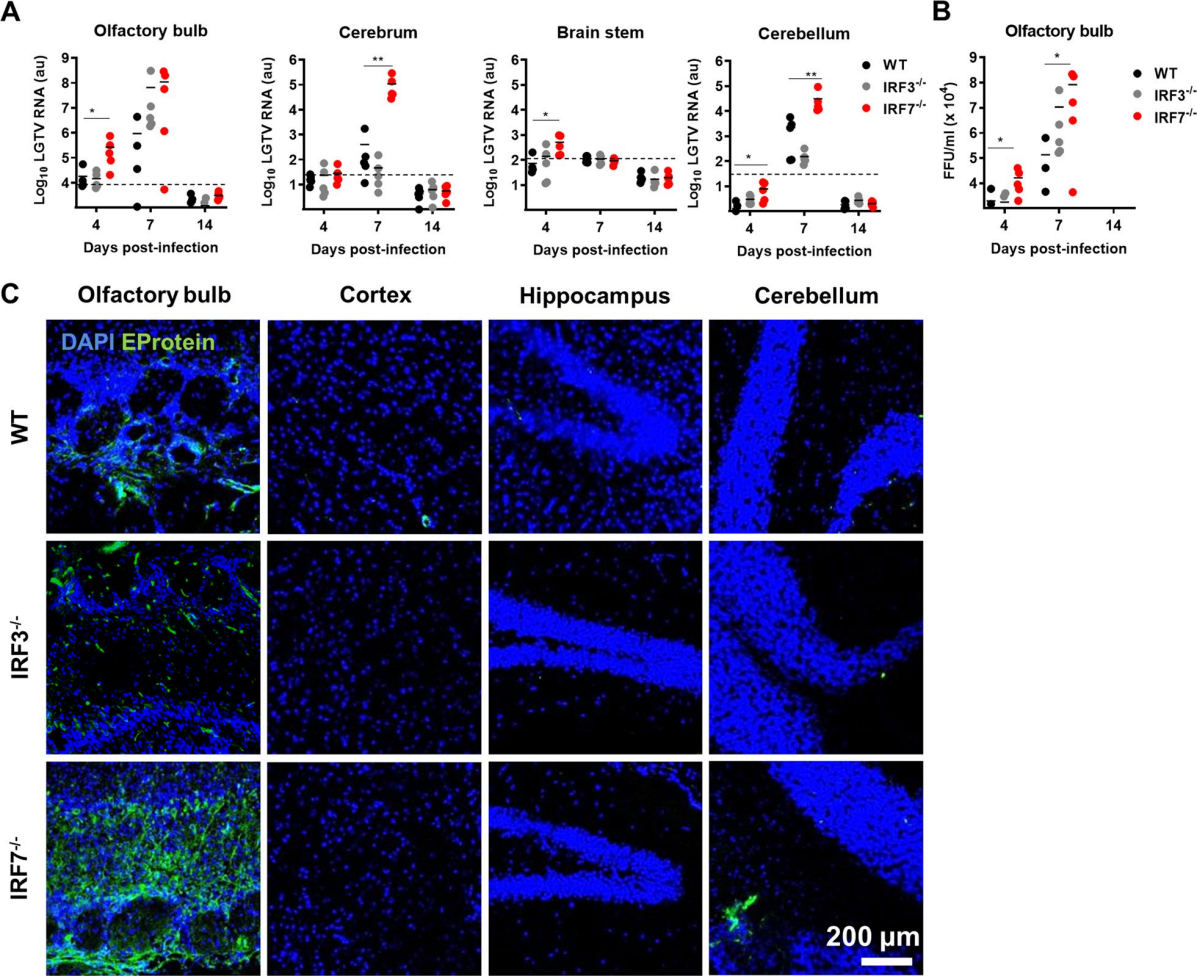
IRF-7 limits viral replication in the periphery and the CNS. WT, *Irf3*^*−/−*^ and *Irf7*^*−/−*^ mice were intraperitoneally infected with 10^4^ FFU of LGTV. Viral burden was determined by quantitative RT-PCR in the different brain regions (**A**) or by plaque assay in the olfactory bulb (**B**) (*n* = 10). The dotted line represents the detection limit. Asterisks indicate statistical significance calculated by Student’s *t* test, *p < 0.05. **C** Representative pictures of immunohistological analysis of LGTV-E protein in the olfactory bulb, cortex, hippocampus and cerebellum at 7 days post-infection (*n* = 5) [LGTV E protein (green), DAPI (blue)]. Magnification is represented by scale bar, 200 µm

Since LGTV is a neurotropic virus, we determined viral replication and spread of the virus into the CNS [14]. Viral RNA was quantified during the course of infection in different brain parts by quantitative real-time RT-PCR (Fig. 2A). Increased viral RNA levels were detectable in different CNS regions in *Irf7*^*−/−*^ mice 4 days post-infection. The levels further increase in all brain parts of *Irf7*^*−/−*^ animals 7 days post-infection compared to WT and *Irf3*^*−/−*^ mice. The highest values were determined in the olfactory bulb. In the cerebrum and cerebellum, the viral load increased significantly in Irf7^−/−^ mice. Analysis of viral burden by plaque assay in the olfactory bulb shows that determination of viral burden by quantitative real-time RT-PCR and plaque assay is comparable.

To investigate the impact of IRF7 on viral replication, distribution and spread to specific CNS structures we performed immunohistological analysis (IHC) of different brain regions on day 7 post-infection (Fig. 2B). The limited sensitivity of the antibody does not allow quantification, but can be used to determine the distribution of the viruses in the tissue. High numbers of viral E-protein positive cells were detected in the glomerular layer of the olfactory bulb in *Irf7*^*−/−*^ mice. Viral E-protein-positive cells could also be detected in the olfactory bulb of WT and in *Irf3*^*−/−*^ mice to a lower extent. Other brain areas were essentially free of E-protein-positive cells except for a small area in the cerebellum of *Irf7*^*−/−*^ mice. Thus, together with our RT-PCR results, the IHC confirmed that the lack of IRF7 allows uncontrolled viral replication of LGTV in the CNS, especially in the olfactory bulb.

### Cytokines and IFN response

**Fig. 3:**
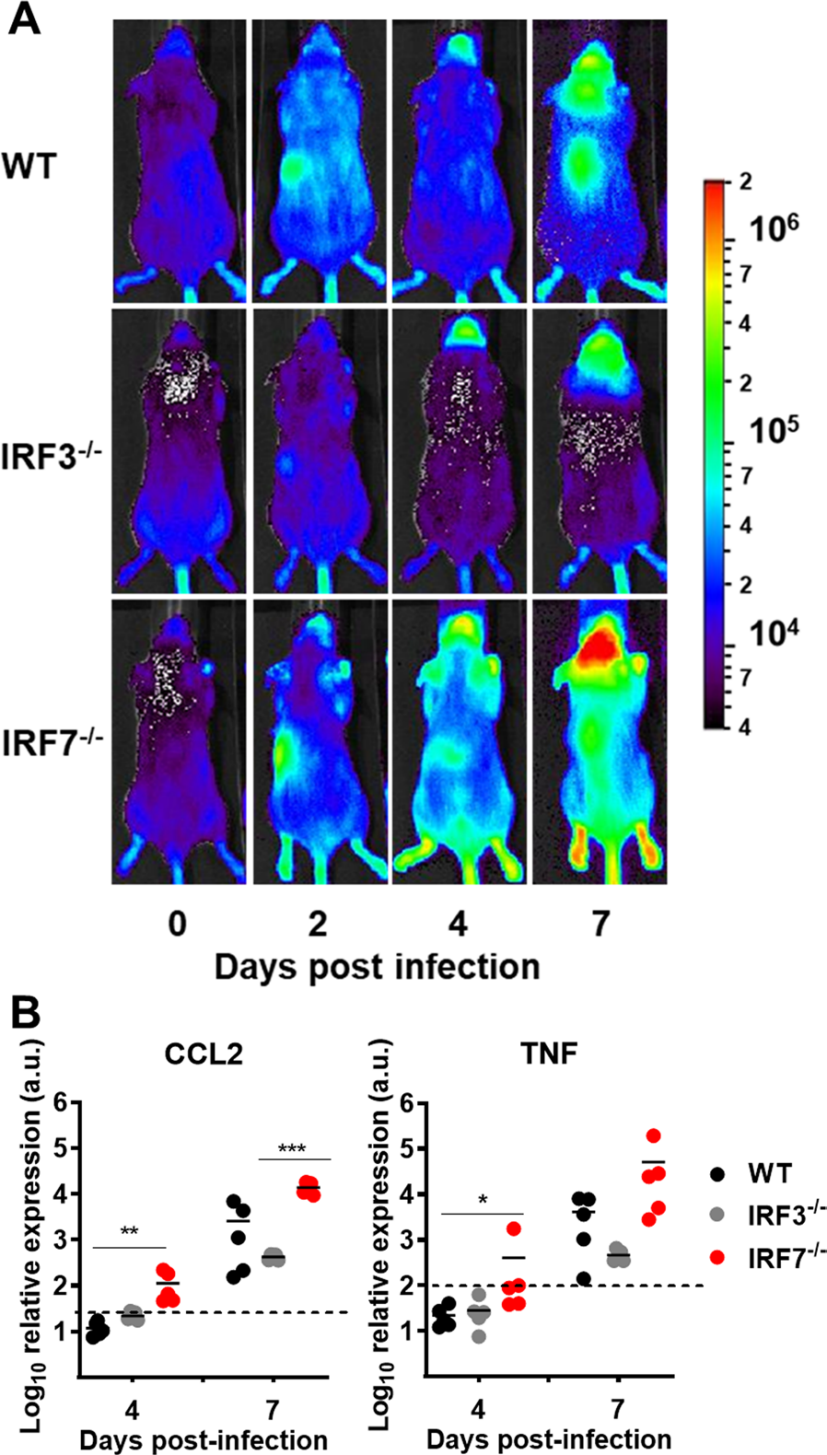
IRF7 deficiency increases cytokine response in the brain. WT, *Irf3*^*−/−*^ and *Irf7*^*−/−*^ mice (*n* ≥ 10) were intraperitoneal infected with 10^4^ FFU of LGTV. **A** Level of IFNβ induction in LGTV-infected *IFNβ*^+*/Δluc*^ mice. Shown is whole body imaging of *Ifnβ*^+*/Δluc*^ WT, *Irf3*^*‑/‑*^ or *Irf7*^*‑/‑*^ reporter mice. Mice were imaged before treatment (0 h) and over time as indicated; one representative mouse is shown in dorsal position. The scale indicates the number of photons (p) measured per s per cm^2^ per steradian (sr). **B** Cytokines CCL2 and TNFα were determined by quantitative RT-PCR in the brain. The dotted line represents the detection limit. Asterisks indicate statistical significance calculated by Student’s *t* test, *p < 0.05, **p < 0.01, ***p < 0.001

We have previously shown that the local IFN-I response is essential to control viral replication in the brain. To determine localization and kinetics of IFN-β induction not only in the periphery but also in the brain, we took advantage of a luciferase-based IFN-β reporter mouse strain (*Ifnβ*^+*/Δluc*^) [25] to image whole body IFN-β induction in vivo (Fig. 3A). As assumed from serum IFN-I levels, only low induction of IFN-β was detectable in the periphery upon systemic infection with neurotropic LGTV. However, we found that the expression of IFN-β was upregulated in the CNS of *Irf7*^*−/−*^* Ifnβ*^+*/Δluc*^ mice 7 days post-infection compared to WT *Ifnβ*^+*/Δluc*^ and *Irf3*^*−/−*^* Ifnβ*^+*/Δluc*^ animals. Thus, IFN-I expression in the brain is regulated by a different IRF7-independent mechanism.

Given the high amount of IFN-β in the brain, we examined the expression of proinflammatory cytokines in the brain. We observed that expression of inflammatory chemokine CCL2 and cytokine TNF-α (Fig. 3B) increased in the brain of *Irf7*^*−/−*^ during the course of infection.

### The loss of IRF7 leads to enhanced recruitment of immune cells to the CNS

**Fig. 4:**
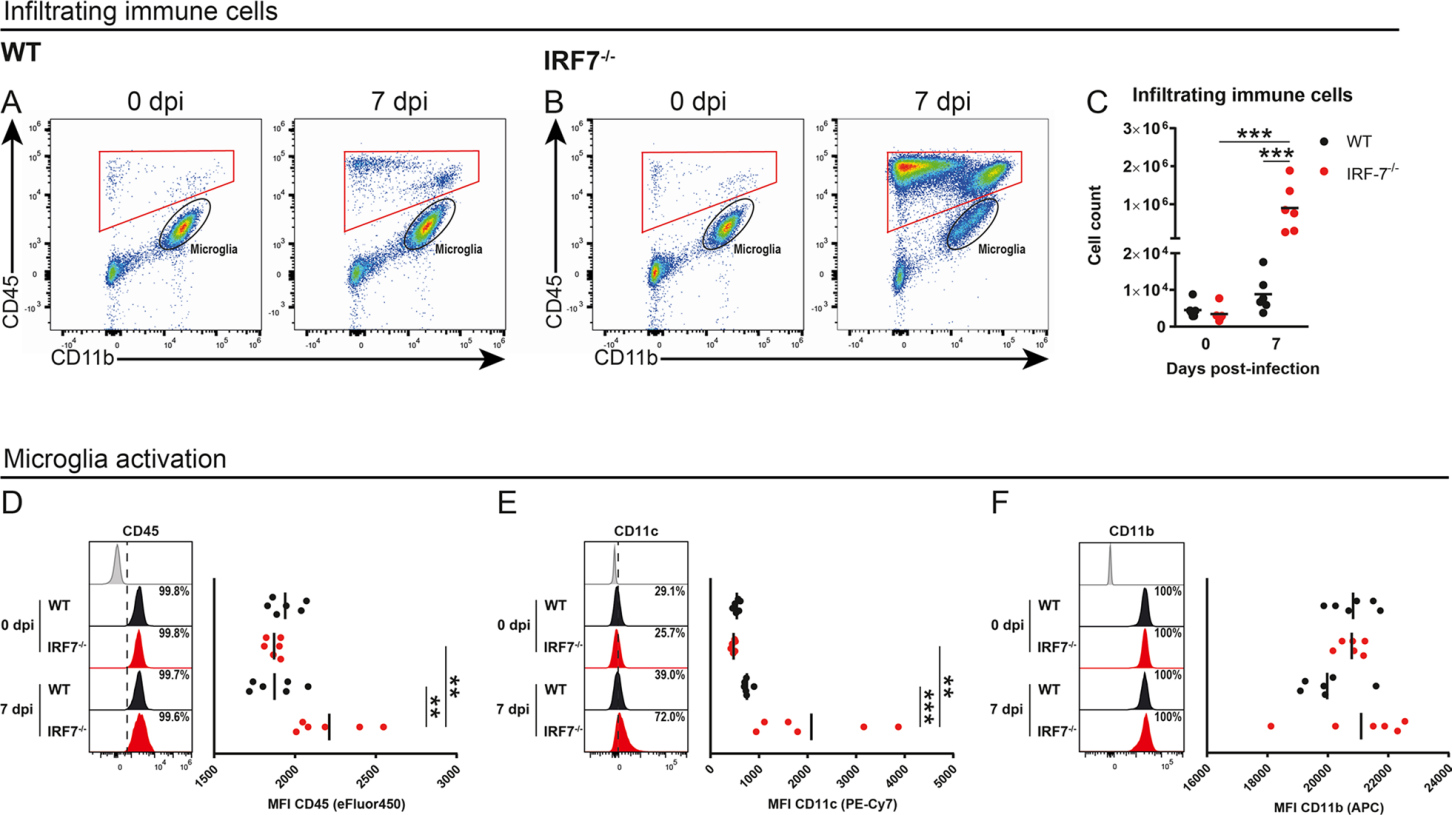
IRF7 deficiency leads to inflammatory response and infiltration of immune cells into the brain. WT and *Irf7*^*−/−*^ mice were infected intraperitoneally with 10^4^ FFU LGTV (*n* = 6) and immune cells were isolated from brains for flow cytometric characterization on day 7 post-infection. Upon infection with LGTV, brain immune cells were manually gated based on their size and granularity in the forward scatter and side scatter light (FSC/SSC) and then separated from dead cells and doublets (not shown). Subsequently, infiltrating immune cells were distinguished from brain-resident microglial cells based on their high expression levels of CD45 and CD11b, respectively (red gate; **A**, **B**). Compared to WT littermates, infected *Irf7*^*−/−*^mice displayed high numbers of infiltrating immune cells in the brain parenchyma 7 dpi (**C**). Furthermore, the surface expression levels of CD45 (**D**), CD11c (**E**), and CD11b (**F**) by microglial cells were assessed. Representative histograms with dashed vertical lines show the frequency (with standard error of the mean) of microglial cells from WT (black tint) and *Irf7*^*−/−*^ mice (red tint) expressing the respective surface marker in comparison to the corresponding FMO control (grey tint) on day 0 and 7 post-infection. Scatter plots display the median fluorescence intensity (MFI) of microglia and highlight an increased activation state of these cells in *Irf7*.^*−/−*^ mice upon infection with LGTV. Data are represented as mean and asterisks indicate statistical significance calculated by 2-way ANOVA with Tukey’s post hoc test, **p< 0.01, ***p < 0.001

Since expression of IFNs and proinflammatory cytokines is often associated with neuroinflammation, we performed flow cytometric analysis of infiltrating immune cells and brain-resident microglia cells (Fig. 4A, B). While only low numbers of peripheral CD45^+^ immune cells were observable in WT mice upon LGTV infection, we found a substantial increase of infiltrating cells in the brains of *Irf7*^*−/−*^ mice on day 7 post-infection (Fig. 4C). In addition, phenotypic characterization of brain-resident microglia from *IRF7*-deficient mice indicated an upregulation of the surface markers CD45 and CD11c when compared to WT mice, translating into an overall increased activation state of these cells upon LGTV infection (Fig. 4D–F).

### Recruited peripheral immune cells are highly diverse

**Fig. 5:**
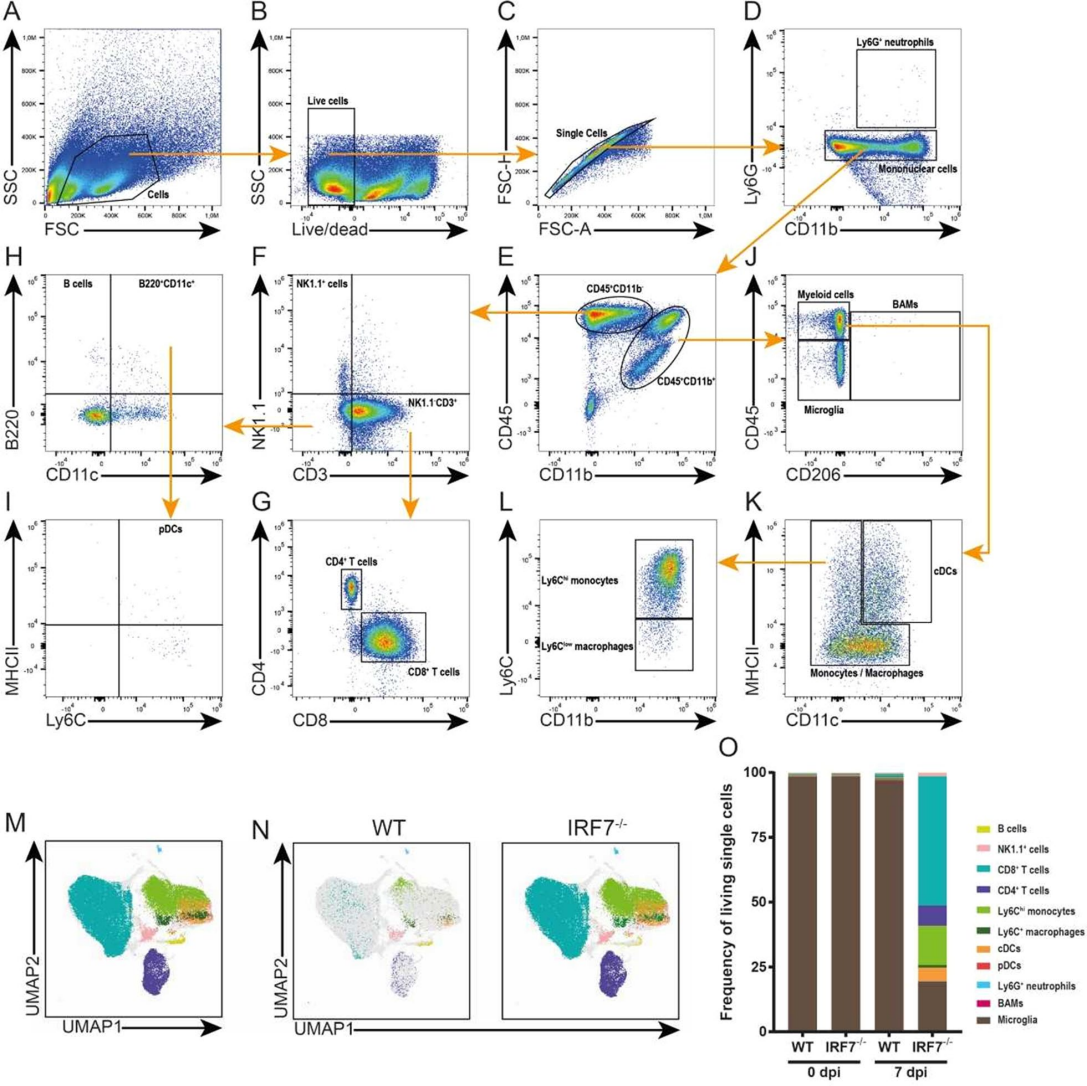
High diversity of infiltrating immune cells in brains of IRF7-deficient mice upon LGTV infection. WT and Irf7^−/−^ mice were infected intraperitoneally with 10^4^ FFU LGTV (*n* = 6) and immune cells were isolated from brains for flow cytometric characterization. A representative gating strategy (A-K). FSC and SSC gating of immune cells (**A**). Life cell selection (**B**) and separation from doublets (**C**). Neutrophil granulocytes Ly6G + CD11b + (**D**), differentiation of remaining mononuclear cells by CD45 and CD11b expression (**E**). Identification of NK1.1^+^ and NK1.1^−^CD3^+^ cells within CD45^+^CD11b^−^ population (**F**). NK1.1^−^CD3^+^ cells were gated for CD4 and CD8 (**G**). Remaining NK1.1^−^CD3^−^ cells from (**F**) were separated in B220^+^CD11c^−^ B cells (**H**). B220^+^CD11c^+^ cells (**H**) which were further assessed for MHC class II (MHCII) and Ly6C expression show plasmacytoid dendritic cells (pDCs) (**I**). CD45^+^CD11b^+^ cells from (**E**) were gated for CD45 and CD206 separating brain-resident microglia from border-associated macrophages (BAMs) and infiltrating myeloid cells (**J**). MHCII and CD11c expression of myeloid cells to distinguish conventional dendritic cells (cDCs) and monocytes/macrophages (**K**). Monocytes and macrophages characterization by CD11b and Ly6C expression: Ly6C^hi^ inflammatory monocytes, Ly6Clow macrophages (**L**). Clustering of Infiltrating immune Uniform Manifold Approximation and Projection for Dimension Reduction (UMAP), constructing a framework of cells while preserving the global structure of sample. Cells within clusters were subsequently identified by overlaying previously gated populations (**M**) and compared between groups of LGTV-infected WT and Irf7^−/−^ mice on day 7 post-infection (**N**). Stacked bar charts display the overall frequency of immune cell subsets within the brains of mice on day 0 and 7 post-infection (**O**)

To obtain a more differential insight into the composition of infiltrating immune cells observed in *IRF7*-deficient mice, we utilized a manual gating approach and identified the presence of several major immune cell subsets, such as Ly6G^+^ neutrophils, CD4^+^ and CD8^+^ T cells, and Ly6C^hi^ inflammatory monocytes (Fig. 5A–K). In parallel, we subjected the flow cytometry datasets to unsupervised computational clustering by Uniform Manifold Approximation and Projection for Dimension Reduction (UMAP), and found a major overlap between formed clusters and our manually defined cell populations (Fig. 5M). Furthermore, the comparison of clusters and cell numbers from LGTV-infected WT and *Irf7*^*−/−*^ mice 0 and 7 days post-infection reconfirmed our previous findings of prominent differences in the composition of infiltrating immune cells between the four experimental groups (Fig. 5N, O).

### IRF7 deficiency leads to changes in the cellular tropism of LGTV

**Fig. 6:**
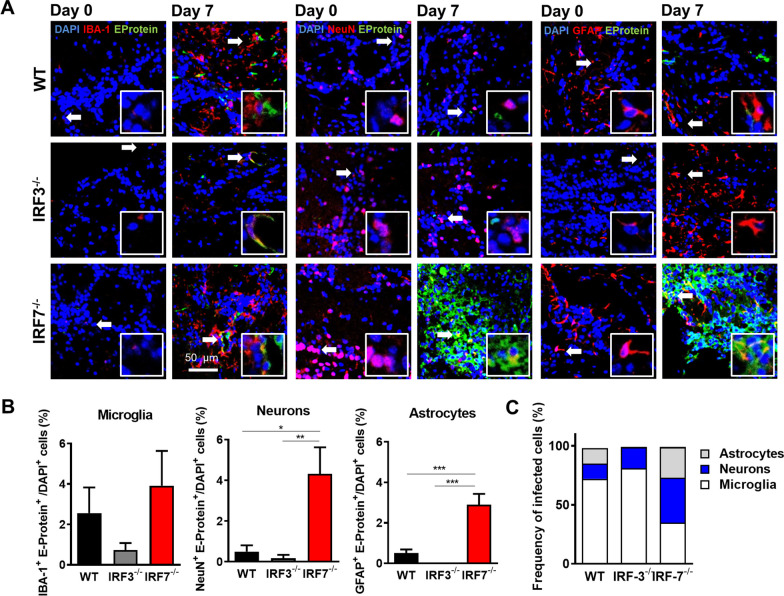
IRF7 deficiency leads to differences in cellular tropism of LGTV. WT, and *Irf7*^*−/−*^ mice were infected intraperitoneally with 10^4^ FFU of LGTV. Brains were isolated for immunohistological analysis 7 days post-infection. **A** Representative pictures of IBA-1 (red), NeuN (red), GFAP (red) and, LGTV-E protein (green), and DAPI (blue). Scale bar is shown as 50 μm. **B** Quantification of LGTV-positive, NeuN^+^, GFAP^+^, and IBA-1^+^ cells in the glomerular layer of the olfactory bulb in infected mice, 7 days post-infection (*n* = 5). Asterisks indicate statistical significance calculated by Student’s *t* test, *p < 0.05, **p< 0.01, ***p < 0.001. **C** Proportion of microglia, neurons and astrocytes from infected cells

We recently demonstrated that neurons are the main target cells for viral replication in the brain upon LGTV infection [14, 19]. To examine which cell type harbors viral antigens in the CNS, tissue sections from WT and IRF7-deficient mice were stained with antibodies against TBEV E protein and cell type-specific markers (Fig. 6A). As expected we observed a larger proportion of LGTV-positive cells in the IHC analysis of the olfactory bulb in IRF7-deficient mice when compared to WT and *Irf3*^*−/−*^*animals*. The co-staining with DAPI, IBA-1, NeuN, and GFAP indicated few infected cells which showed overlap of neurons, astrocytes and microglia markers with viral E protein expression. Quantification of viral E-protein-positive cells revealed, that loss of IRF3 and IRF7 has no impact on the number of infected microglia. However, in neurons and astrocytes loss of IRF7 leads to significantly elevated numbers of infected cells (Fig. 6B). Calculation of the proportion of cell types in the infected cells revealed that neurons and astrocytes are affected by the loss of IRF7. Thus, IRF7 protects resident cells of the CNS, namely astrocytes and neurons, from LGTV infection.

### IRF3 and IRF7 mediate a brain-specific antiviral response

**Fig. 7:**
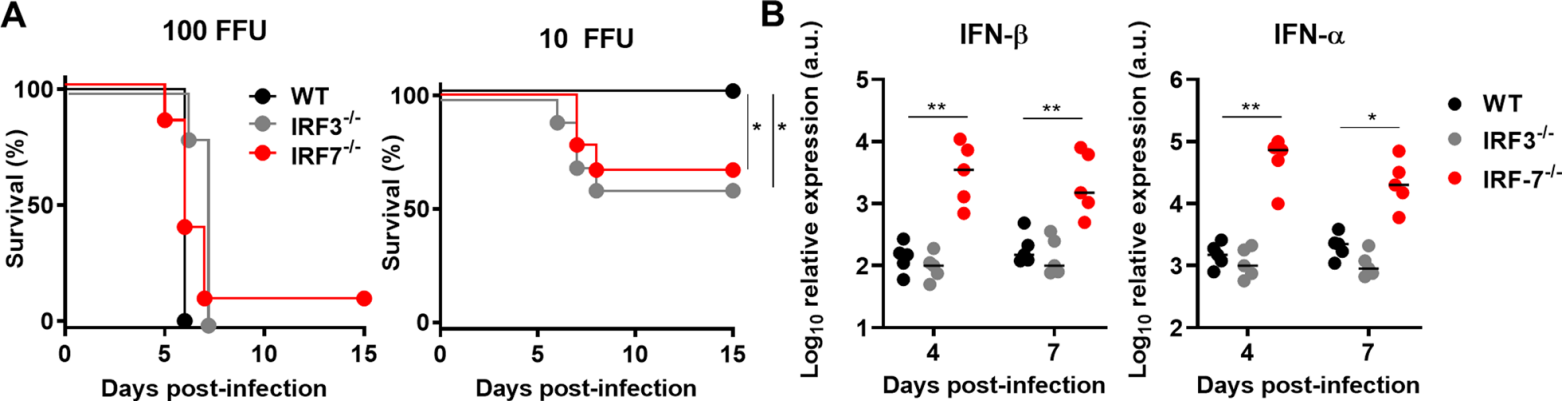
Local antiviral role of IRF7 upon LGTV infection in the brain. WT, and *Irf7*^*−/−*^ mice were infected intracranially with LGTV. **A** Kaplan–Meier curve of mice infected with 10^2^ FFU (*n* = 5) or 10 FFU (*n* = 5). Data are cumulative from two independent experiments. Asterisks indicate statistical significance calculated by Mann–Whitney test, *p < 0.05. **B** IFN-β and IFN-α determination by quantitative RT-PCR in the brain of mice infected intracranial by 10 FFU with LGTV. Asterisks indicate statistical significance calculated by Mann–Whitney test (**A**) or Student’s *t* test (**B**), *p < 0.05, **p < 0.01. Determination of viral titers (**B**) and IFN-I production (**C**) from primary hippocampal neuron or whole-brain astrocytes cultures. **D** Primary astrocytes were transfected with increasing amounts of poly(I:C). Supernatants were harvested 24 h post-transfection and IFN-I was determined by a bioassay. **E** Survival analysis of LGTV-infected *Ifnβ*^*−/∆luc*^ and *Irf7*^*−/−*^* Ifnβ*^*−/∆luc*^ mice. Asterisks indicate statistical significance calculated by Mantel–Cox test (**A**, **E**) and 2-way ANOVA (**B**–**D**), *p < 0.05, ***p<0.001. Results are representative for at least two independent experiments

The control of viral infections in the CNS is dependent on different factors, e.g., the extent of the viremia, the number of virus particles entering the brain or local immune responses. To determine the impact of local antiviral responses in the brain, we infected WT, *Irf3*^*−/−*^ and *Irf7*^*−/−*^ mice intracranially (i.c.) with 10 FFU or 100 FFU LGTV and determined the survival (Fig. 7A). Nearly all animals succumbed to infection with symptoms within a mean survival time of 5 to 7 days post-infection when infected i.c. with 100 FFU of encephalitis. Upon i.c. infection with 10 FFU, 100% of WT but only 60–70% of the *Irf3*^*−/−*^ and *Irf7*^*−/−*^ animals survived. This indicates that both IRF3 and IRF7 are crucial for the formation of a regionally restricted antiviral status in the CNS independent of peripheral immune responses.

### Astrocytes mediate a cell-specific intrinsic antiviral response upon LGTV infection

**Fig. 8:**
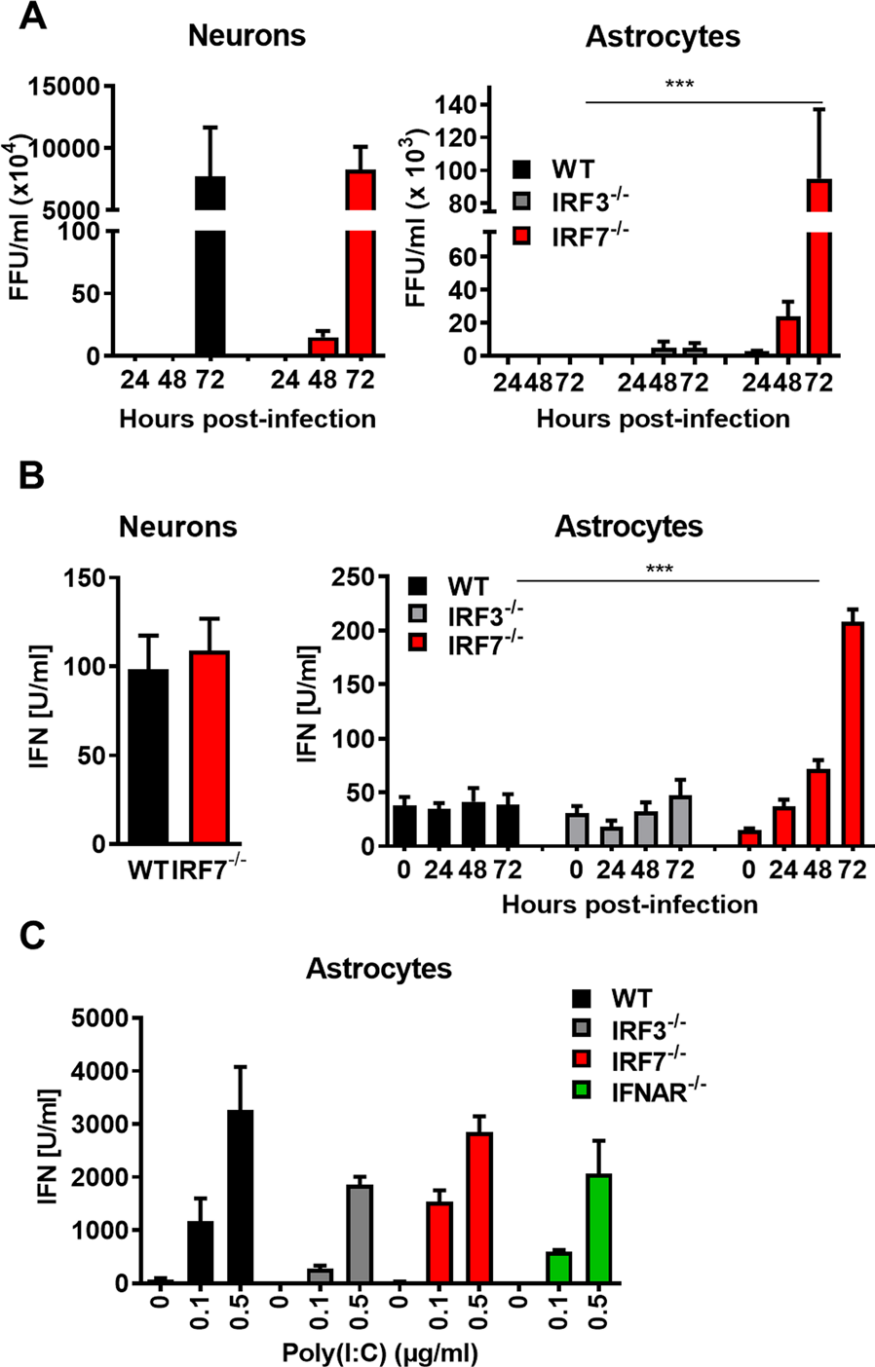
Cell type-specific antiviral role of IRF7 upon LGTV infection in the brain. Determination of viral titers (**A**) and IFN-I production (**B**) from primary hippocampal neuron or whole-brain astrocytes cultures. **C** Primary astrocytes were transfected with increasing amounts of poly(I:C). Supernatants were harvested 24 h post-transfection and IFN-I was determined by a bioassay. Asterisks indicate statistical significance calculated by Student’s *t* test, ***p<0.001. Results are representative for at least two independent experiments

Since neurons and astrocytes are the main target cells of LGTV in *Irf7*^*−/−*^ mice we further wanted to determine the role of IRF7 in cell-autonomous control of LGTV replication in these cell types. We isolated primary neural cells from the hippocampus and brain astrocytes and performed infection studies (Fig. 8A). Primary neural hippocampus cells are highly susceptible to LGTV and infection with 0.01 MOI leads to high viral titers. Surprisingly, IRF7 deficiency did not result in elevated viral growth. In astrocytes, no virus replication was detectable in WT and only partly in *Irf3*^*−/−*^ cells. In contrast, IRF7 deficiency was associated with increased virus replication and viral titer was tenfold higher compared to neurons (Fig. 8A). These data suggest that IRF7 is critical to limit viral replication in astrocytes, whereas neurons are vulnerable to LGTV infection independent of the tested genotype. However, elevated viral levels in IRF7-deficient neurons in vivo (Fig. 7B) but not in vitro (Fig. 8A) suggest a mechanism of an IRF7-dependent intercellular network that transfers antiviral signals to neurons in vivo.

### Loss of IRF7 in astrocytes leads to a strong IFN-I response, which is independent of the IFNAR/IRF7-mediated positive feedback

IRF7 is a critical regulator of IFN-I induction and amplification. To investigate which cells of the CNS produced IFN-I upon LGTV infection we isolated supernatants from infected primary neurons and astrocytes and quantified IFN-I levels by bioassay. Infected neuronal cells from WT and *Irf7*^*−/−*^ mice secreted comparable amounts of IFN-I into the supernatant (Fig. 8B). WT astrocytes failed to induce IFNs in response to LGTV but surprisingly *Irf7*^*−/−*^ astrocytes mounted a high IFN-I production (Fig. 8B). These data suggest, that in the absence of IFN-I amplifier IRF7, astrocytes are able to mount high levels of IFN-I.

High viral replication correlates with increased quantities of PAMPs, which results in the induction of IFN-I [26]. To determine if the high amount of viral RNA may be responsible for the increased production of IFN-I in astrocytes, we transfected astrocytes with increasing amounts of poly (I:C), a synthetic equivalent of viral RNA (Fig. 8C). Astrocytes from all genotypes were tested and showed a concentration-dependent production of IFN-I. This indicates, that astrocytes can mount high levels of IFN-I expression independent of the IRF7/IFNAR-mediated amplification of IFN-I response by an intensified stimulation of PRR signaling.

### IRF7-independent induction of IFNβ protects mice from lethal infection

**Fig. 9:**
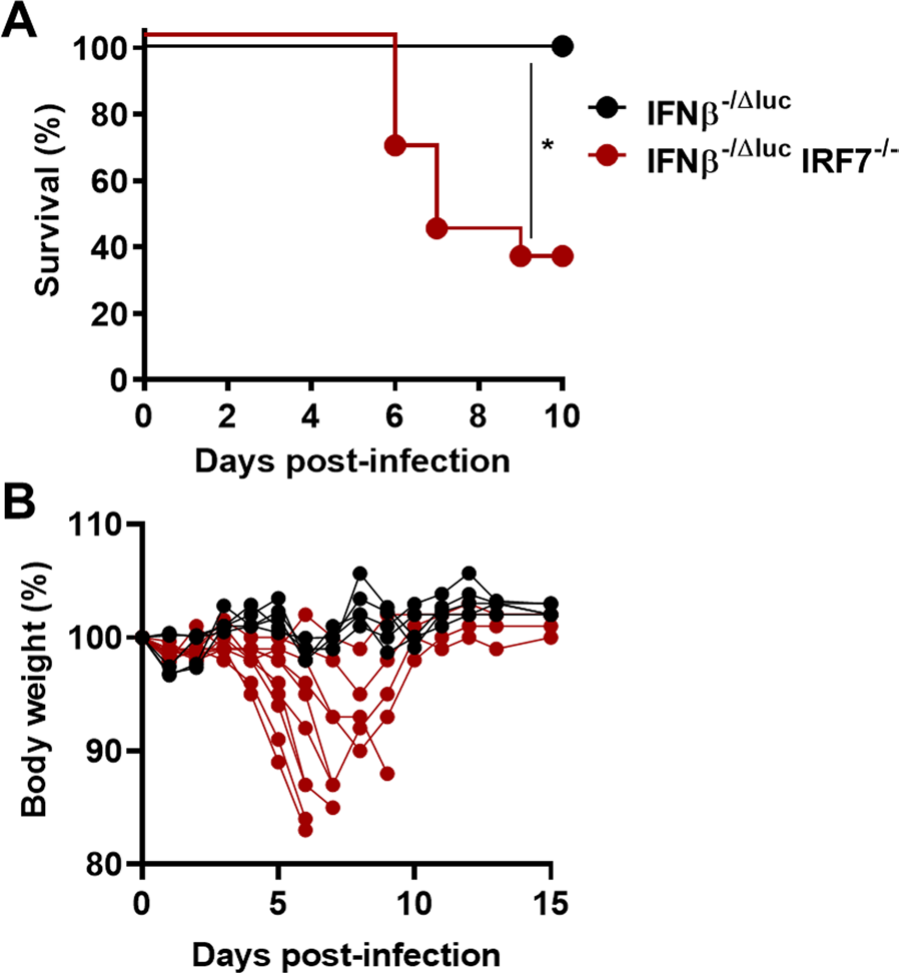
IFNβ protects IRF7-deficient mice in LGTV infection. *Ifnβ*^*−/∆luc*^ and *Irf7*^*−/−*^* Ifnβ*^*−/∆luc*^ mice were intraperitoneally infected with 104 FFU of LGTV. **A** Kaplan–Meier curve. **B** Body weight during course of infection. Asterisks indicate statistical significance calculated by Mann–Whitney test, *p<0.05. Results are representative for at least two independent experiments

Finally, we were interested in determining whether IRF7-independent induction of elevated IFN-I amounts mediates a protective effect in mice upon LGTV infection. For this, we infected *Irf7*^*−/−*^*Ifnβ*^*−/−*^ mice, which are not able to produce IFN-β, with LGTV (Fig. 9). Approximately 70% of the mice succumbed to LGTV infection, indicating that the high amount of IFN-I produced upon infection in *Irf7*^*−/−*^ mice protects the mice from death although the massive IFN-I production was not able to protect neurons from infection.

## Discussion

We have previously described that the antiviral activity of IFN-I during neurotropic infection is important in both, the periphery to control viral replication and spread to the CNS, and locally in the central nervous system to directly inhibit viral replication [14]. How this IFN-I induction is regulated in the CNS has remained unclear so far. In the current study, we investigated the impact of IRF3 and IRF-7, which are important transcription factors involved in the regulation of IFN-Is. Using *Irf3*^*−/−*^ and *Irf7*^*−/−*^ mice, we demonstrated a cell type-specific role of IRF7 in the antiviral response in brain-resident cells during neurotropic infection. We detected that IRF7 is dispensable for the antiviral response and IFN-I induction in neurons. In contrast, astrocytes rely on IRF7 to orchestrate an intrinsic antiviral response. Still in the absence of IRF7, a high expression of IFN-I by astrocytes occurs independently on the IFNAR/IRF7-mediated positive feedback loop.

IRF3 and IRF7 are important transcription factors regulating the expression of IFN-I and ISGs [27, 28]. Although they belong to the same family of interferon regulatory factors and share a homolog DNA-binding domain, IRF3 and IRF7 bind to distinct but overlapping target genes. Further regulation of IRF7 is more strictly controlled than IRF3 [29]. Therefore, they are able to fine-tune the antiviral response which may explain their non-redundant role in the pathogenesis during viral infections [30, 31]. In some virus infections, the antiviral effect is independent of IFN-I, IRF3, IRF5 or IRF7, and deficient mice survive the infection. Furthermore, an IRF1-dependent antiviral response mediates the essential antiviral response independent of IFN-I [21, 32, 33].

The importance of IRF3 and IRF7 for the survival of viral infections is controversial. Whereas IRF3-deficient mice mostly survived neurotropic infections with normal IFN-I induction in the periphery, IRF7 is needed for proper peripheral IFN-I responses and survival of several virus infections, e.g., with Encephalomyocarditis virus (EMCV), Herpes simplex virus (HSV-1), WNV or Oropouche virus (ORO) [11, 34, 35]. In some cases synergistic effects of IRF3, IRF7 or IRF5 are necessary for protective IFN-I responses [36], since viruses developed pleiotropic mechanism to circumvent the antiviral response.

In our model, IRF3 and IRF7-deficient mice show none or only minor symptoms of sickness and survived a systemic infection, although peripheral IFN-I response is dampened in *Irf7*^*−/−*^ mice. In accordance with other studies, the loss of IRF7 leads to abolished IFN-I concentration in the serum and production by BMDCs and at least, limited secretion by BMDMs upon LGTV infection. In contrast, the loss of IRF3 had no impact on the circulating IFN-I level which leads to reduced viremia in WT and *Irf3*^*−/−*^ mice in comparison to *Irf7*^*−/−*^ mice. We recently showed that the IFN-I antiviral response is essential for the survival of LGTV infections, since mice lacking other distinct components of the IFN-I pathway like MAVS and IFNAR succumb to infection quite rapidly [14, 19]. Thus, a specific role of IRFs in an organ- or cell type-specific induction of antiviral responses is likely.

In contrast to the well-established role of IRF7 in the periphery, very little is known about its specific function in the CNS. Here, we show that even though these animals were protected from systemic infections, they succumbed to local LGTV infection with severe neurological symptoms. Similar results have been achieved upon WNV infections [34]. Differential roles of IFN-I induction were shown recently, whereas induction of IFN-β was highly dependent on MAVS in the olfactory bulb, induction of IFN-β in other brain areas occurs in the absence of MAVS [19]. In addition, regional astrocyte IFN-I signaling differentially regulates blood–brain barrier integrity upon WNV infection [37, 38]. For IRFs differential upregulation of expression was shown in brains upon intracranial infection with LCMV [39].

To determine the underlying mechanism, we analyzed the susceptibility of brain-resident cells to LGTV infection and the subsequent IFN-I induction in more detail. Hippocampal neurons were highly susceptible to LGTV infection in vitro independent of the presence of IRF7. We did not detect any differences by loss of IRF7 in their ability to control viral replication or secrete IFN-I (Fig. 6E), in contrast to comparable studies in cortical neurons infected with WNV [34]. Thus, inhibition of virus replication and induction of IFN-I is not dependent on IRF7. However, reduced viral replication in WT but not in IRF7-deficient neurons from LGTV infection suggests an extrinsic antiviral response in vivo.

Microglia cells carry out an important role in maintaining CNS health and play an important role in disease pathology. IRF7 expression in microglia is associated with a shift from a pro-inflammatory M1 phenotype to an anti-inflammatory M2 phenotype during spinal cord injury [40]. It was shown that both infected and non-infected microglia upregulate IRF7 upon VSV infection, but only infected microglia are able to induce IFN-β expression [41]. Our experiments revealed that loss of IRF7 expression does not lead to increased viral infection of microglia.

Astrocytes, another brain-resident cell type, support neuronal cells for instance by production of growth factors and synthesis of immunomodulatory substances [42, 43]. It has been published that astrocytes and to a certain extent neurons can contribute to the local IFN-I response in the brain, especially in the olfactory bulb [18, 44]. Astrocytes were identified as the main producers of IFN-I and thus confer to an extrinsic antiviral response even if they are not productively infected [17, 18, 45]. Upon TBEV infection astrocytes show a biphasic IFN-β induction that initially depends on MAVS and later on MyD88/TRIF signaling [46]. We have shown that astrocytes are in an antiviral status [47], which could explain why they are mainly abortively infected. The maintenance of this low expression of ISGs might be dependent on IRF7. This could explain that the loss of IRF7 results in a changed cellular tropism and an increased number of infected astrocytes upon LGTV infection in vivo. Although a high constitutive expression of IRF7 has not been identified in the CNS [44], this low constitutive expression might be sufficient for induction or efficient priming of antiviral gene expression. A similar mode of action was observed upon loss of IFNAR in MEFs, *IFNAR*^*−/−*^ cells were highly susceptible to viral infections compared to cells treated with IFN-I neutralizing antibodies. This might be explained by a reduced basal expression of ISGs upon the sustained loss of IFNAR.

We demonstrated that in contrast to WT astrocytes, LGTV infection of IRF7-deficient astrocytes leads to a high IFN-I production. IFN-I signaling in astrocytes not only limits early viral spread in the CNS, but also promotes antiviral IFN-γ response [48]. The high IFN-I production in astrocytes was rather unexpected since a high IFN-I production is either dependent on an IFNAR/IRF7-mediated positive feedback or a strong stimulation of MyD88/TRIF-adapted TLR stimulation in pDCs. Virus infection studies with VSV and New Castle disease virus (NDV), or stimulation with artificial double-stranded RNA poly(I:C) can induce only minor IFN-I response in the absence of this positive feedback loop [10, 49]. This suggests another mechanism of high-level IFN-I induction in astrocytes, since a stimulation with poly(I:C) leads to high IFN-I induction regardless of the presence of IRF7 or IFNAR. The exact mechanism of how IFN I expression was induced in astrocytes was unclear. Our data suggest that high IFN-I induction in astrocytes is mediated by enhanced stimulation of RIG-I/MAVS signaling.

In summary, we conclude, that even though IRF7 is dispensable for the survival of systemically infected mice, it plays an important role in the restriction of viral replication and the induction of local IFN-I responses. We have underlined cell- and tissue-specific functions for IRF7 in the periphery and the CNS. Moreover, our data suggest that a loss of IRF7 promotes viral replication, supporting the proinflammatory status of the brain, and indicating a local IFN-I response by astrocytes. These findings further highlight the molecular mechanisms of the early protective antiviral immune response, uncovering novel strategies for therapeutic intervention against neurotropic viral pathogens.

## Data Availability

Data are available from the corresponding author upon reasonable request.

## Abbreviations

CNS: Central nervous system
FFU: Focus formation unit
i.c.: Intracranial
IFN-I: Type I interferon
i.p.: Intraperitoneal
IRF3: Interferon regulatory factor 3
IRF7: Interferon regulatory factor 7
ISG: Interferon stimulated gene
LGTV: Langat virus
PAMPs: Pathogen associated molecular pattern
pDC: Plasmacytoid dendritic cell
PRRs: Pathogen recognition receptors
RIG-I: Retinoic acid-inducible gene
RLRs: Retinoic acid-inducible gene-like receptor
TBEV: Tick-borne encephalitis virus
TLR: Toll-like receptor
WNV: West Nile virus
VSV: Vesicular stomatitis virus
